# The power of reflective practice: evaluating the impact of a psychoeducation and reflective practice group for surgical nursing staff and health care assistants in a trauma centre

**DOI:** 10.1192/bjo.2021.515

**Published:** 2021-06-18

**Authors:** R Harrison

**Affiliations:** Avon and Wiltshire Mental Health Partnership NHS Trust

## Abstract

**Aims:**

To offer a psychoeducation and reflective practice group for nursing staff (NS) and health care assistants (HCAs) working on a Trauma and Orthopaedics Ward in Southmead Hospital, Bristol. To explore the staff experience of having a reflective space, and how this impacted on their attitudes and knowledge and confidence in psychiatric presentations.

**Background:**

Reflective practice can raise the quality and consistency of nursing care, but it is not part of everyday culture and practice. Southmead Hospital is a trauma centre and the surgical NS and HCAs care for multiple patients following self-harm or suicide attempts. They report at times not having the mental health knowledge and confidence to appropriately manage patients on the ward and are at high risk of occupational stress and burnout. Our mental health liaison team (MHLT) identified this need and offered to provide a space to address these concerns and evaluate the impact of this intervention.

**Method:**

After liaising with the ward manager, I developed and provided a fortnightly forty-minute psychoeducation and reflective practice group for NS and HCAs on one Trauma and Orthopaedic ward in Southmead Hospital. Topics were rotated and included suicidal ideation, self-harming behaviour, mind and body link, the stress -vulnerability model and verbal aggression.

The staff were asked to complete anonymous paired pre-and post-course questionnaires about their attitudes and confidence regarding mental health difficulties. This questionnaire included both quantitative components (e.g. 1–5 Likert scales) and qualitative components (free text boxes) which were analysed and coded accordingly.

**Result:**

Quantitative results showed that staff felt it was important to learn about mental health conditions and have a reflective space. Their confidence and knowledge improved in understanding and managing psychiatric presentations. Qualitative results revealed several common themes – (i) Space; staff valued a protected, structured, safe space, (ii) Relationships: staff valued sharing with colleagues and supporting each other, (iii) Sharing and learning; staff valued a space to think about patient's formulations, discuss common experiences, express their own emotions and learn from each other and (iv) Psychoeducation; the staff welcomed ideas of ways to communicate with patients and specific skills to use on the wards.

**Conclusion:**

Trauma and Orthopaedic NS and HCAs perceived a range of benefits from participating in a psychoeducation and reflective practice group. Further research is required to evaluate whether reflective practice groups help to reduce staff burnout and can change the ward ethos to improve the patient experience.

